# Fine-Tuning of Alkaline Residues on the Hydrophilic Face Provides a Non-toxic Cationic α-Helical Antimicrobial Peptide Against Antibiotic-Resistant ESKAPE Pathogens

**DOI:** 10.3389/fmicb.2021.684591

**Published:** 2021-07-15

**Authors:** Xudong Luo, Xiangdong Ye, Li Ding, Wen Zhu, Pengcheng Yi, Zhiwen Zhao, Huanhuan Gao, Zhan Shu, Shan Li, Ming Sang, Jue Wang, Weihua Zhong, Zongyun Chen

**Affiliations:** ^1^Institute of Biomedicine and Hubei Key Laboratory of Embryonic Stem Cell Research, College of Basic Medicine, Hubei University of Medicine, Shiyan, China; ^2^Hubei Key Laboratory of Wudang Local Chinese Medicine Research, Hubei University of Medicine, Shiyan, China; ^3^Department of Clinical Laboratory, Dongfeng Hospital, Hubei University of Medicine, Shiyan, China; ^4^Central Laboratory of Xiangyang No. 1 People’s Hospital, Hubei University of Medicine, Shiyan, China; ^5^Department of Rehabilitation Medicine, Taihe Hospital, Hubei University of Medicine, Shiyan, China

**Keywords:** ESKAPE pathogens, antibiotic resistance, cationic α-helical antimicrobial peptide, hydrophilic face, lysine vs arginine, hemolytic activity, *in vivo* efficacy

## Abstract

Antibiotic-resistant ESKAPE pathogens (*Enterococcus faecium*, *Staphylococcus aureus*, *Klebsiella pneumoniae*, *Acinetobacter baumannii*, *Pseudomonas aeruginosa*, and *Enterobacter species*) has become a serious threat to public health worldwide. Cationic α-helical antimicrobial peptides (CαAMPs) have attracted much attention as promising solutions in post-antibiotic era. However, strong hemolytic activity and *in vivo* inefficacy have hindered their pharmaceutical development. Here, we attempt to address these obstacles by investigating BmKn2 and BmKn2-7, two scorpion-derived CαAMPs with the same hydrophobic face and a distinct hydrophilic face. Through structural comparison, mutant design and functional analyses, we found that while keeping the hydrophobic face unchanged, increasing the number of alkaline residues (i.e., Lys + Arg residues) on the hydrophilic face of BmKn2 reduces the hemolytic activity and broadens the antimicrobial spectrum. Strikingly, when keeping the total number of alkaline residues constant, increasing the number of Lys residues on the hydrophilic face of BmKn2-7 significantly reduces the hemolytic activity but does not influence the antimicrobial activity. BmKn2-7K, a mutant of BmKn2-7 in which all of the Arg residues on the hydrophilic face were replaced with Lys, showed the lowest hemolytic activity and potent antimicrobial activity against antibiotic-resistant ESKAPE pathogens. Moreover, *in vivo* experiments indicate that BmKn2-7K displays potent antimicrobial efficacy against both the penicillin-resistant *S. aureus* and the carbapenem- and multidrug-resistant *A. baumannii*, and is non-toxic at the antimicrobial dosages. Taken together, our work highlights the significant functional disparity of Lys *vs* Arg in the scorpion-derived antimicrobial peptide BmKn2-7, and provides a promising lead molecule for drug development against ESKAPE pathogens.

## Introduction

Bacterial resistance to traditional antibiotics is a serious threat to human health. The majority of antibiotic-resistant infections are caused by the ESKAPE pathogens (*Enterococcus faecium*, *Staphylococcus aureus*, *Klebsiella pneumoniae*, *Acinetobacter baumannii*, *Pseudomonas aeruginosa*, and *Enterobacter species*) (Pendleton et al., 2013). Recently, the WHO listed these bacteria as the key pathogens against which novel antimicrobial agents are urgently needed (Tacconelli et al., 2018).

Cationic α-helical antimicrobial peptides (CαAMPs) are typically short defensive peptides that are widely distributed in various animals, such as scorpions (Harrison et al., 2014), wasps (Konno et al., 2016) and frogs (Patocka et al., 2019), and many of them showed direct antimicrobial activity against various pathogens. CαAMPs are amphiphilic molecules with a high content of positively charged residues (Lys and Arg) and hydrophobic residues in their sequences (Fjell et al., 2011; Mahlapuu et al., 2016; Ciumac et al., 2019). CαAMPs can fold into amphiphilic α-helices upon insertion into negatively charged bacterial plasma membranes, where they form various kinds of lipophilic pores that induce membrane disruption, cellular metabolite leakage, and eventually bacterial death (Fjell et al., 2011; Mahlapuu et al., 2016; Ciumac et al., 2019). From this mode of action, CαAMPs kill bacteria rapidly and have a much lower tendency to produce bacterial resistance, which makes CαAMPs attractive alternatives to conventional antibiotics.

Although hundreds of CαAMPs were originally identified, significant drawbacks, such as strong hemolytic activity and *in vivo* inefficacy, have hindered their pharmaceutical development (Mahlapuu et al., 2016; Ghosh et al., 2019; Magana et al., 2020). In recent studies, many attempts have been made to improve the performance of CαAMPs. These strategies involve re-engineering natural CαAMP sequences by site-directed mutation (Ahmad et al., 2009; Irazazabal et al., 2016; Li et al., 2016; Jiang et al., 2020), fragmentation (Luo et al., 2021) or chemical modification (Zarina and Nanda, 2014; Nayak et al., 2018; Mourtada et al., 2019; Mwangi et al., 2019; Li et al., 2020), computational approaches based on natural templates and statistical analysis (Loose et al., 2006; Nagarajan et al., 2018, 2019), or designing *de novo* sequences by using simple alkaline *vs* hydrophobic amino acid combinations (Deslouches et al., 2005, 2013; Hu et al., 2011; Lakshmaiah Narayana et al., 2020). However, partly due to the complexity of the structure-activity relationships, which are derived from the sequence diversity of the peptides, progress in CαAMP optimization is still very limited and few CαAMPs have successfully achieved FDA approval (Greber and Dawgul, 2017; Ghosh et al., 2019; Torres et al., 2019).

BmKn2 is a 13-mer naturally occurring CαAMP that was identified in the scorpion *Mesobuthus martensii* Karsch. This peptide possesses strong hemolytic activity and merely shows antimicrobial activity against gram-positive bacteria, such as *S. aureus*. BmKn2-7, which has lower hemolytic activity and a broadened antimicrobial spectrum, is a mutant of BmKn2 (Cao et al., 2012). For the design of optimized AMP, we first investigated the structural basis that determines the distinct functional performances of BmKn2 and BmKn2-7. Our results revealed that an increase in the number of alkaline residues (Lys and Arg) on the hydrophilic face can result in reduced hemolytic activity and a broadened antimicrobial spectrum, but the difference in the Lys *vs* Arg combination may significantly influence the hemolytic activity of the peptide. Based on this, we designed a series of BmKn2-7-derived peptides that share the same hydrophobic face but contain different Lys *vs* Arg combinations on the hydrophilic face. Strikingly, we found that replacement of Arg with Lys on the hydrophilic face does not influence the antimicrobial activity of the peptide but can significantly reduce its hemolytic activity. The peptide BmKn2-7K, in which all of the Arg residues were replaced with Lys residues on the hydrophilic face, showed the lowest hemolytic activity.

The antimicrobial potential of BmKn2-7K was evaluated thereof. *In vitro* experiments demonstrated that BmKn2-7K exhibits potent antimicrobial activity *via* a membrane-lytic mechanism against a series of clinically isolated antibiotic-resistant ESKAPE pathogens, including gram-positive bacteria such as methicillin-resistant *S. aureus* (MRSA), methicillin-resistant *S. epidermidis* (MRSE), *E. faecalis* and *E. faecium*, and gram-negative bacteria such as extended-spectrum β-lactamase (ESBL)-producing *E. coli*, carbapenem-resistant (CRE) or multidrug-resistant (MDR) *P. aeruginosa*, CRE and MDR *A. baumannii* and CRE *K. pneumoniae*. *In vivo* experiments showed that BmKn2-7K is non-toxic at antimicrobial dosages and displays potent antibacterial efficacy against both penicillin-resistant *S. aureus* and CRE- and MDR *A. baumannii*. In short, our work highlights the significant functional disparity of Lys vs Arg in the scorpion-derived antimicrobial peptide BmKn2-7, and provides new clues for the development of novel antimicrobials against drug-resistant ESKAPE pathogens.

## Materials and Methods

### Peptide Synthesis and Bioinformatic Analysis

All peptides were synthesized and purified by ChinaPeptides Corporation (Shanghai, China) by solid-phase methods using standard N-9-fluorenylmethyloxycarbonyl (Fmoc) chemistry (Colombo, 1982). The molecular weights were measured by electrospray ionization mass spectrometry (ESI-MS, TripleTOF 5600, AB Sciex, United States). Peptide purity (>95%) was determined by reverse-phase high-performance liquid chromatography (RP-HPLC) with a Kromasil 100-5C18 column (4.6 mm × 250 mm) at 220 nm at a flow rate of 1.0 ml/min, using a linear water/acetonitrile gradient that contained 0.1% trifluoroacetic acid. Peptides were stored as lyophilized powders before use. The theoretical molecular weights, isoelectric points (pI) and grand average of hydropathicity (GRAVY) were calculated online using the ProtParam tool^1^ (Gasteiger et al., 2003). The helical-wheel plots, net charge and hydrophobic moments were calculated online using the HeliQuest server^2^ (Gautier et al., 2008). The three-dimensional structure projections were predicted online using the I-TASSER server^3^ (Yang and Zhang, 2015).

### Circular Dichroism

The secondary structural content of the peptide was measured by circular dichroism (CD) spectroscopy. Peptides were dissolved in either distilled H_2_O or 2,2,2-trifluoroethanol (TFE, Aladdin) solutions to achieve a concentration of 150 μg ml^–1^. The CD spectra (λ_190–250 *nm*_) were obtained at 25°C on a J-820 spectropolarimeter (Jasco, Tokyo, Japan) using a quartz cell with a 1 mm light path. Three scans were recorded for each sample. The mean residue molar ellipticity was calculated from the original CD data by the equation as described (Yang et al., 2019):


$$\begin{array}{c} \theta =\left(\theta_{obs}*1000\right)/\left(c*l*n\right) \end{array}$$

where θ is the mean residue molar ellipticity, θ_*obs*_ is the observed ellipticity corrected relative to the buffer, *c* is the peptide concentration (mM), *l* is the path length (mm), and *n* is the number of residues of the peptide.

### Hemolytic Activity Determination

Fresh human red blood cells (hRBCs) were collected in a sterile borosilicate glass tube covered with sodium citrate as the anticoagulating agent. Then, the cells were washed three times with 0.9% sodium chloride via centrifugation (1,000 × *g*, 4°C, 5 min) and prepared in a sterile 96-well polypropylene plate to achieve a final concentration of 4% (v/v). Two-fold dilutions of the peptides were prepared in 0.9% sodium chloride to 100 μl and mixed with equal volumn of hRBC suspension to final peptide concentrations ranging from 12.5 to 200 μg ml^–1^. After co-incubation at 37°C for 1 h, the samples were centrifuged (2,000 × *g*, 4°C, 20 min), and the supernatant (100 μl) was taken for optical density (OD_540_) measurements (*A*_*sample*_). The hRBCs assayed with 0.9% sodium chloride (*A*_*blank*_) or 1% Triton X-100 (*A*_*positive*_) were applied as 0% and 100% hemolysis, respectively. Three independent experiments were performed and the percent hemolysis was calculated according to the following equation (Yin et al., 2012; Li et al., 2016):


$$\begin{array}{c} \text{Hemolysis}\left(\%\right)=100*\left(A_{\text{sample}}-A_{\text{blank}}\right)/\left(A_{\text{positive}}-A_{\text{blank}}\right) \end{array}$$

### Bacterial Strains

The bacteria used in this study included the standard strains *S. aureus* ATCC29213 and ATCC25923, *E. faecalis* ATCC29212, *E. coli* ATCC25922 and ATCC35218, *A. baumannii* ATCC19606, *K. pneumoniae* ATCC700603 and *P. aeruginosa* ATCC27853, these strains were were purchased from the China Center of Type Culture Collection (CCTCC). The antimicrobial activities of the peptides were also determined against clinically isolated strains *S. aureus* 4188, 9124, 1176, *S. epidermidis* 9092, 6943, 888, *E. faecalis* 901, *E. faecium* 898, *E. coli* 2678, 2687, *K. pneumoniae* 9126, *P. aeruginosa* 9014, 9042, and *A. baumannii* 906, 13012, 13079, 9068, these strains were acquired from Taihe Hospital, Hubei University of Medicine. The drug sensitivities of the clinical isolates to the antibiotics were determined by the Kirby-Bauer test (Chinnambedu et al., 2020).

### Minimum Inhibitory Concentration Determination

The MIC determination was performed using the broth dilution method according to the guidelines of the Clinical and Laboratory Standards Institute (CLSI) (Cao et al., 2012; Mourtada et al., 2019). Briefly, the bacterial cells were incubated in Mueller-Hinton broth (MHB, Oxoid) at 37°C and 150 rpm, after overnight incubation, the bacteria were transferred to new broth until the exponential phase of growth (OD_630_ = 0.4). After that, the bacteria were diluted with fresh MHB to 160 μl and prepared in a sterile 96-well polypropylene plate to achieve a final concentration of 6.25 × 10^5^ colony forming units (cfu) per ml. Two-fold dilutions of the peptides in 0.9% sodium chloride (40 μl) were added to each well to achieve the final peptide concentrations ranging from 1.25 to 80 μg ml^–1^. After co-incubation at 37°C for 20 h, each MIC was determined by optical density (OD_630_) measurements as the minimum concentration of peptide with no detectable bacterial growth. All experiments were repeated at least three times.

To study the influence of physiological ions on the antimicrobial activities of the peptides, The MICs of the peptides were also determined against the standard bacterial strains cultured in MHB containing various salts at their physiological concentrations (150 mM NaCl, 4.5 mM KCl, 1 mM MgCl_2_, 2 mM CaCl_2_, 6 μM NH_4_HCO_3_, and 4 μM FeCl_3_) (Ma et al., 2016).

### Time-Killing Kinetics

Time-dependent killing abilities of the peptide were evaluated against *S. aureus* ATCC29213. Briefly, the bacteria were cultured in MHB at 37°C and 150 rpm until the log phase of growth. Then, the bacteria (1 × 10^6^ cfu ml^–1^) were co-incubated with a series of 1, 2 or 4 × MIC peptide in a sterile 96-well plate at 37°C and 150 rpm. Aliquots of the samples were taken at different time points (0, 5, 15, 30, and 60 min), diluted appropriately, and spread on Mueller-Hinton agar. The surviving colonies were determined after the cells were cultured overnight. The bacteria treated with 4 × MIC melittin was used as a positive control.

### Membrane Permeabilization

Membrane permeabilization caused by the peptide was determined by propidium iodide (PI, Thermo Fisher) uptake assays as described previously (Luo et al., 2021). Briefly, mid-log phase bacteria *S. aureus* ATCC29213 was collected by centrifugation (6,000 × *g*, 4°C) and washed three times with phosphate-buffered saline (PBS). Next, the bacterial cells were diluted to OD_630_ = 0.1 and prepared in a sterile 96-well polypropylene plate. After mixing with 2 μM PI, a series of concentrations of the peptide (1, 2 or 4 × MIC) were added into each well, and fluorescence was immediately recorded on a Molecular Devices SpectraMax i3x (excitation wavelength: 535 nm, emission wavelength: 617 nm). All experiments were repeated three times. The data were normalized against the fluorescence values of the bacteria treated with 4 × MIC melittin which was used as a positive control.

### Membrane Depolarization

The effect of the peptide on membrane depolarization was monitored by measuring the fluorescence of DiSC_3_-5 (Sigma-Aldrich), a membrane potential sensitive probe (Suzuki et al., 2003; Venkatesh et al., 2017). Briefly, mid-log phase bacteria *S. aureus* ATCC29213 were harvested by centrifugation (6,000 × *g*, 5 min), washed twice with N-2-hydroxyethylpiperazine-N-2-ethane sulfonic acid (HEPES) buffer (5 mM HEPES, 20 mM glucose, 100 mM potassium chloride, pH 7.4), and diluted with the same buffer to achieve an OD_630_ of 0.05. Then the bacteria were incubated with DiSC_3_-5 (0.4 μM) in the dark for 90 min to achieve a stable reduction in fluorescence. After the addition of a series of concentrations of the peptides (1, 2 or 4 × MIC), fluorescence was immediately measured on a microplate reader (Molecular Devices SpectraMax i3x) with an excitation wavelength of 622 nm and an emission wavelength of 670 nm. All experiments were repeated three times. The data were normalized against the fluorescence values of the bacteria treated with 4 × MIC melittin which was used as a positive control.

### Transmission Electron Microscopy

For transmission electron microscopy (TEM) observation, *S. aureus* ATCC29213 were cultured in MHB to achieve an OD_630_ = 0.2. Then, the bacteria were treated with 1 × MIC peptide for 0.5 h (150 rpm and 37°C). After that, the bacterial cells were harvested (10,000 × g, 4°C, 5 min) and fixed with 2.5% glutaraldehyde (Sigma-Aldrich) (Ma et al., 2016). The morphologies of the bacteria were observed by using an FEI Tecnai G2 20 TWIN transmission electron microscope.

### Cytotoxicity

The cytotoxicity of the peptide was determined against mouse fibroblast cells L929 and human embryonic kidney cell 293T (HEK293T). Briefly, the cells were seeded at a density of 8,000 cells per well in a 96-well plate and cultured for 24 h in Dulbecco’s Modified Eagle Medium (DMEM, Gibco) supplemented with 10% fetal bovine serum (FBS, Gibco), 2 mM L-glutamine, 100 U ml^–1^ penicillin, 100 mg ml^–1^ streptomycin (1% P/S, Invitrogen^TM^), and at 37°C in a 5% of CO_2_ atmosphere. Then, different concentrations of the peptide (0, 10, 20, 30, and 40 μg ml^–1^) were added into each well and co-incubated with the cells for 24 h. After 2 h incubation with 10 μM 2-(2-methoxy-4-nitrophenyl)-3-(4-nitrophenyl)-5-(2,4-disulfophenyl)-2H-tetrazolium (CCK-8, Yeasen, China), the cytotoxicity of the peptide was determined by measuring the optical density at 450 nm (Wu et al., 2014; Li et al., 2020).

### *In vivo* Systemic Toxicity

Healthy ICR mice (male, 6–8 weeks old, ∼30 g) were used to determine the *in vivo* systemic toxicity of the peptide (Chen et al., 2013). The mice were randomly divided into six groups (*n* = 10). Five cohorts were intraperitoneally injected with a single dose of 10, 20, 40, 80, and 160 mg kg^–1^ body weight of the peptide dissolved in PBS. The mice injected with PBS were used as control. The survival of mice was inspected for 7 days. All surviving mice were euthanized at the end of the experiments.

For histopathological examination, each group of ICR mice (*n* = 6) was injected intraperitoneally with PBS or with a single dose of 40 mg kg^–1^ body weight of the peptide. At 1, 2, and 7-day post-injection, the mice were euthanized, and the liver, kidney and spleen were collected and fixed with 4% formaldehyde for haematoxylin and eosin staining.

### Peritonitis Models

Healthy ICR mice (male, 6–8 weeks old, ∼30 g) were used to evaluate the *in vivo* antimicrobial activity of the peptide. Overnight-cultured bacteria were transferred to new broth and cultured to the log phase of growth. Then, the bacteria were collected and washed three times with PBS by centrifugation (6,000 × *g*, 4°C, 5 min). The mice were intraperitoneally injected with a single dosage of 5 × 10^7^ cfu of *S. aureus* 4188 (or 6.25 × 10^7^ cfu of *A. baumannii* 906) suspended in PBS to establish the mouse peritonitis models. For each bacteria, one cohort (*n* = 10) was euthanized at 0.5 h post-infection and the bacterial load in the peritoneal fluid was determined to ensure the development of the peritonitis model. Other cohorts (*n* = 10) were treated intraperitoneally with a single dosage of the peptide or treated with PBS which was used as a placebo. The survival of the mice was recorded for 7 days.

To confirm the antimicrobial efficacy of the peptide, the bacterial loads in the peritoneal fluid of the mice treated with the peptide were determined. Briefly, the infected mice were randomly sub-grouped into three cohorts (*n* = 6). One cohort of the mice was euthanized at 0.5 h post-infection, which was used as control. Other cohorts of the mice were treated with the peptide or PBS at the same time point. After 4 h treatment, the mice were euthanized and soaked in 75% ethanol for 5 min. Then, the peritoneum was washed by injecting 5 ml of PBS, followed by gentle massaging and peritoneal fluid extraction. The fluid was diluted appropriately and spread on Mueller-Hinton agar. The bacterial loads were determined after overnight incubation.

### Statistic Analysis

The data obtained were analyzed using the software GraphPad Prism 5.0. The statistical variances between each group were evaluated by one-way ANOVA, followed by the Tukey’s *post hoc* test. Significant variances are marked with asterisks (^∗^*P* < 0.05, ^∗∗^*P* < 0.01 and ^∗∗∗^*P* < 0.001).

## Results

### Increasing the Number of Lys or Arg Residues on the Hydrophilic Face Reduces the Hemolytic Activity of BmKn2 and Broadens Its Antimicrobial Spectrum

**FIGURE 1:**
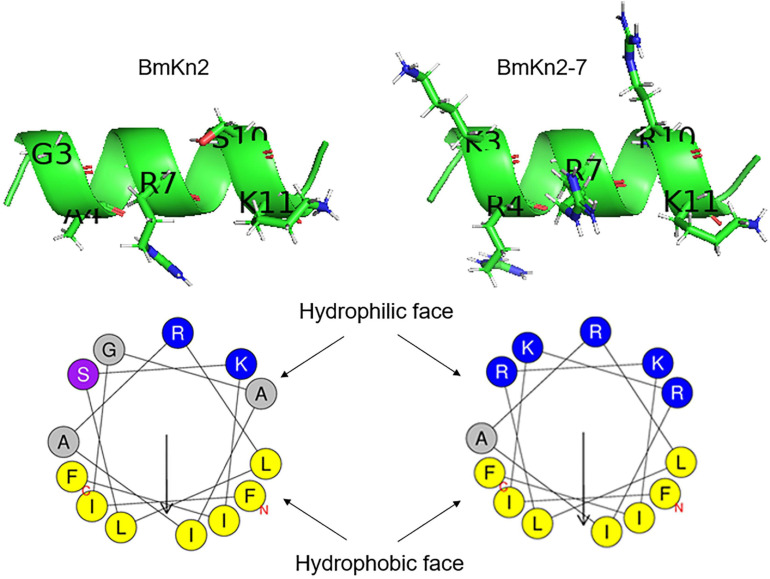
Structural variations between the scorpion cationic α-helical antimicrobial peptide (CαAMP) BmKn2 and BmKn2-7. The three-dimensional structures and the helical-wheel plots were obtained by using the I-TASSER and HeliQuest servers, respectively. Residues of the hydrophilic faces are marked with one letter abbreviations and side chains are shown in the ribbon structures. In the helical-wheel plots, residues marked in blue and yellow represent positively charged alkaline and hydrophobic amino acids, respectively. Residue marked in purple represents Ser. Residues marked in gray represent Gly and Ala.

In order to design AMP with optimized performance, we investigated BmKn2, a naturally occurring AMP which was reported to exhibit good antimicrobial activity against gram-positive bacteria but exhibits strong hemolytic activity, and its analog BmKn2-7, which displays better antimicrobial and lower hemolytic activities (Cao et al., 2012). As shown in Figure 1 and Table 1, no compositional variation on the hydrophobic face of the peptides was observed between the BmKn2 and BmKn2-7, but Gly^3^, Ala^4^ and Ser^10^ on the hydrophilic face of BmKn2 were replaced with Lys, Arg and Arg in BmKn2-7, respectively.

**TABLE 1:** Physicochemical parameters of the peptides designed from BmKn2.

Peptides	Sequence	MW^*a*^ (Da) (Cal./Obs.)	z^*b*^	pI^*c*^	GRAVY^*d*^	<μ H>^*e*^
BmKn2	FIGAIARLLSKIF-NH_2_	1447.8/1447.8	3	11.0	1.592	0.824
Kn2(G3K)	FI**K**AIARLLSKIF-NH_2_	1518.9/1519.0	4	11.2	1.323	0.824
Kn2(A4R)	FIG**R**IARLLSKIF-NH_2_	1532.9/1533.0	4	12.0	1.108	0.806
Kn2(S10R)	FIGAIARLL**R**KIF-NH_2_	1516.9/1517.0	4	12.0	1.308	0.806
Kn2(G3K_A4R)	FI**KR**IARLLSKIF-NH_2_	1604.1/1604.1	5	12.0	0.838	0.866
Kn2(G3K_S10R)	FI**K**AIARLL**R**KIF-NH_2_	1588.1/1588.1	5	12.0	1.038	0.873
Kn2(A4R_S10R)	FIG**R**IARLL**R**KIF-NH_2_	1602.0/1602.1	5	12.3	0.823	0.845
BmKn2-7	FIKRIARLLRKIF-NH_2_	1673.2/1673.1	6	12.3	0.554	0.908

*^*a*^Molecular weight (MW): the predicted values (Cal.) were consistant with the measured ones (Obs.).*

*^*b*^Net charge (z).*

*^*c*^Isoelectric point (pI).*

*^*d*^Grand average of hydropathicity (GRAVY).*

*^*e*^Hydrophobic moments (<µH>).*

**FIGURE 2:**
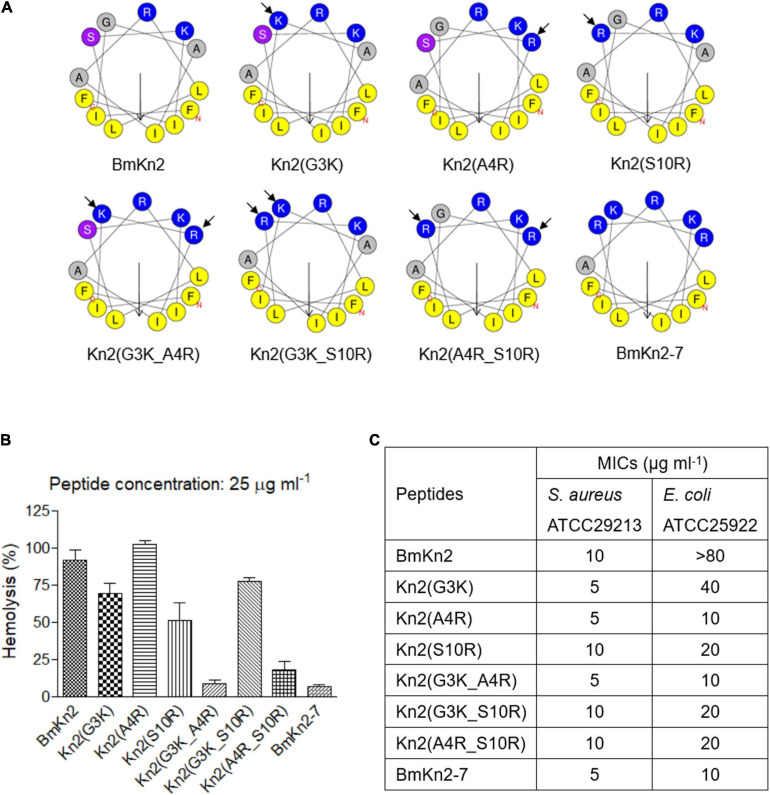
Design, hemolytic and antimicrobial activity analyses of BmKn2 analogs. **(A)** Helical-wheel projections of BmKn2, BmKn2-7 and the designed peptides. **(B)** The percent hRBC hemolysis at 25 μg ml^– 1^ was determined for BmKn2, BmKn2-7 and the designed peptides. The absorbance of the supernatants was measured at 540 nm to evaluate the release of hemoglobin. **(C)** The antimicrobial activities (MIC in μg ml^– 1^) were determined against *Staphylococcus aureus* ATCC29213 and *Escherichia coli* ATCC25922. All experiments were repeated at least three times.

Based on this, six mutants were designed. The first sub-group includes Kn2(G3K), Kn2(A4R) and Kn2(S10R), in which Gly^3^, Ala^4^ and Ser^10^ of BmKn2 was mono-substituted with the corresponding residues in BmKn2-7. The second sub-group includes Kn2(G3K_A4R), Kn2(G3K_S10R) and Kn2(A4R_S10R), in which Gly^3^, Ala^4^ and Ser^10^ in BmKn2 were double-substituted with the corresponding residues in BmKn2-7 (Table 1 and Figure 2A). With an increasing number of alkaline residues (Lys or Arg) on the hydrophilic face, the net charge of these peptides increases step by step from +3 (BmKn2) to +6 (BmKn2-7), and the pI value increases from 11.0 (BmKn2) to 12.3 (BmKn2-7); while the hydrophobic moments slightly increases from 0.824 (BmKn2) to 0.908 (BmKn2-7), the mean hydrophobicity value of the peptides dramatically reduces from 1.592 (BmKn2) to 0.554 (BmKn2-7) (Table 1). To evaluate their antimicrobial and hemolytic activities, all peptides were then synthesized, and the molecular weights of the synthesized peptides were measured by ESI-MS to show consistency with the corresponding predicted peptides (Table 1). By using RP-HPLC, the purities of the synthesized peptides were determined to be greater than 95% (Supplementary Figure 1).

The hemolytic activities of the peptides were determined against hRBCs. Figure 2B shows the hemolytic activities of BmKn2, BmKn2-7 and the mutants at a concentration of 25 μg ml^–1^. Compared to BmKn2 (91.8%), the percent hemolysis of the mono-substituted mutants Kn2(G3K) and Kn2(S10R) were determined to be 69.0% and 50.9%, respectively, but Kn2(A4R) caused 100% hemolysis at this concentration. Interestingly, the percent hemolysis of double-substituted mutants Kn2(G3K_A4R) and Kn2(A4R_S10R), which were determined to be 8.3% and 18.0%, respectively, are very close to that of BmKn2-7 (6.9%). However, the percentage of hemolysis of Kn2(G3K_S10R) was determined to be 77.5% at this concentration, which is much higher than that of Kn2(G3K_A4R) and Kn2(A4R_S10R).

The MICs of the mutants were determined against *S. aureus* (gram-positive) and *E. coli* (gram-negative), two representative bacterial species that were widely used in the previous reports (Cao et al., 2012; Mourtada et al., 2019; Zhao et al., 2021). As shown in Figure 2C, all of them showed potent activity against the gram-positive bacterium *S. aureus* ATCC29213 (MIC: 5–10 μg ml^–1^). Except for BmKn2 (MIC > 80 μg ml^–1^) and Kn2(G3K) (MIC: 40 μg ml^–1^), all the other peptides showed good antimicrobial activity against the gram-negative bacterium *E. coli* ATCC25922 (MIC: 10–20 μg ml^–1^).

Taken together, these results suggest that the increase in the number of alkaline residues on the hydrophilic face of BmKn2 reduces the hemolytic activity of the peptide and broadens its antimicrobial spectrum. Interestingly, different combinations of Lys and Arg on the hydrophilic face may significantly influence the hemolytic activity of the peptide.

### Arg → Lys Substitution on the Hydrophilic Face Significantly Reduces the Hemolytic Activity of BmKn2-7 Without Influencing Its Antimicrobial Activity

**FIGURE 3:**
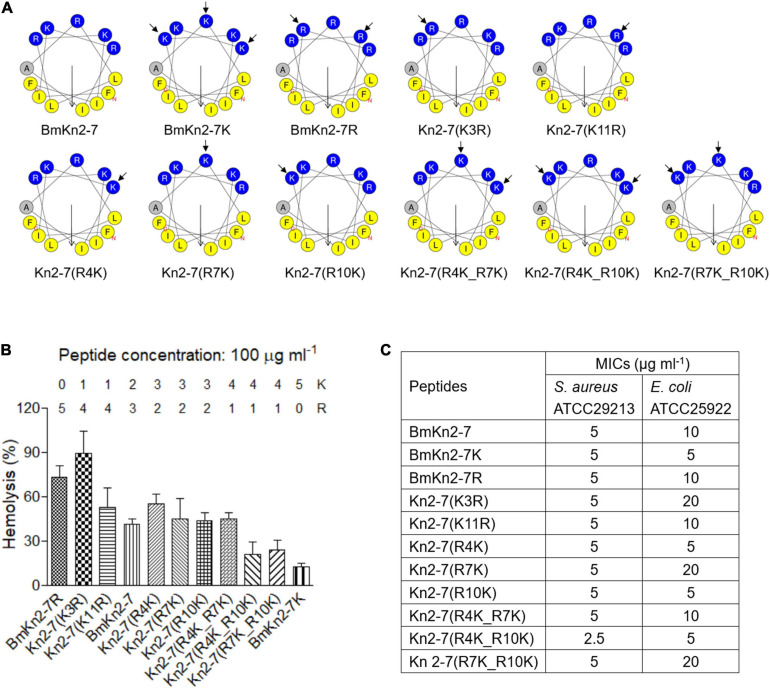
Design, hemolytic and antimicrobial activity analyses of BmKn2-7 analogs. **(A)** Helical-wheel projections of BmKn2-7, BmKn2-7K, BmKn2-7R and the other designed peptides. **(B)** The percent hRBC hemolysis at 100 μg ml^– 1^ was determined for BmKn2-7 and the designed peptides, and the numbers of Arg and Lys residues of each peptide were shown. **(C)** The antimicrobial activity (MIC in μg ml^– 1^) was determined against *S. aureus* ATCC29213 and *E. coli* ATCC25922. All experiments were repeated at least three times.

No optimized AMP better than BmKn2-7 was obtained by the investigations describled above, but the results suggest that alkaline residue composed hydrophilic face is a better choice for the performance of the peptide. Naturally occurring CαAMPs are rich in Lys or Arg with an occurrence frequency of 9.52% and 5.86%, respectively (Wang et al., 2016), but their role in CαAMPs is largely unknown. In order to further optimize BmKn2-7 and obtain deeper knowledge of the effects of Lys and Arg on the hemolytic and antimicrobial activity of CαAMPs, a series of BmKn2-7 mutants were designed. First, all Arg residues on the hydrophilic face of BmKn2-7 were substituted with Lys to give mutant BmKn2-7K, and *vice versa*, BmKn2-7R was obtained (Table 2 and Figure 3A). Then, based on the sequence variations between BmKn2-7 and BmKn2-7K, as well as BmKn2-7 and BmKn2-7R, eight variants were obtained (Table 2 and Figure 3A). With an increasing number of Lys residues on the hydrophilic face, the net charge of these peptides remains constant (+6), which might be own to the incapability of the HeliQuest server to distinguish the difference between the side chain of Lys *vs* Arg; but the pI value decreases from 12.6 (BmKn2-7R) to 10.6 (BmKn2-7K), as predicted by the online ProtParam tool; no obvious variation in amphiphilicity was observed, as indicated with the hydrophobic moment values, but the mean hydrophobicity value of the peptides increases from 0.462 (BmKn2-7R) to 0.692 (BmKn2-7K) (Table 2). All peptides were then synthesized. By ESI-MS, the molecular weights of the synthesized peptides were measured and determined to be consistent with the corresponding predicted ones (Table 2). By using RP-HPLC, the purities of the synthesized peptides were determined to be greater than 95% (Supplementary Figure 2).

**TABLE 2:** Physicochemical parameters of the peptides designed from BmKn2-7.

Peptides	Sequence	MW^*a*^ (Da) (Cal./Obs.)	z^*b*^	pI^*c*^	GRAVY^*d*^	<μ H>^*e*^
BmKn2-7	FIKRIARLLRKIF-NH_2_	1673.2/1673.1	6	12.3	0.554	0.908
BmKn2-7K	FIK**K**IA**K**LL**K**KIF-NH_2_	1589.1/1589.1	6	10.6	0.692	0.905
BmKn2-7R	FI**R**RIARLLR**R**IF-NH_2_	1729.2/1729.2	6	12.6	0.462	0.911
Kn2-7(R4K)	FIK**K**IARLLRKIF-NH_2_	1645.1/1645.1	6	12.0	0.600	0.908
Kn2-7(R7K)	FIKRIA**K**LLRKIF-NH_2_	1645.1/1645.1	6	12.0	0.600	0.907
Kn2-7(R10K)	FIKRIARLL**K**KIF-NH_2_	1645.1/1645.1	6	12.0	0.600	0.907
Kn2-7(R4K_R7K)	FIK**K**IA**K**LLRKIF-NH_2_	1617.1/1617.1	6	11.3	0.646	0.906
Kn2-7(R4K_R10K)	FIK**K**IARLL**K**KIF-NH_2_	1617.1/1617.1	6	11.3	0.646	0.907
Kn2-7(R7K_R10K)	FIKRIA**K**LL**K**KIF-NH_2_	1617.1/1617.1	6	11.3	0.646	0.907
Kn2-7(K3R)	FI**R**RIARLLRKIF-NH_2_	1701.2/1701.2	6	12.5	0.508	0.909
Kn2-7(K11R)	FIKRIARLLR**R**IF-NH_2_	1701.2/1701.2	6	12.5	0.508	0.909

*^*a*^Molecular weight (MW): the predicted values (Cal.) were consistant with the measured ones (Obs.).*

*^*b*^Net charge (z).*

*^*c*^Isoelectric point (pI).*

*^*d*^Grand average of hydropathicity (GRAVY).*

*^*e*^Hydrophobic moments (<µH>).*

The hemolytic activities of the synthesized peptides were determined against hRBCs. The peptides were no longer toxic at 25 μg ml^–1^ and a higher dose was necessary to demonstrate a distinct trend. So, the percent hemolysis of BmKn2-7, BmKn2-7K, BmKn2-7R and the other mutants were comparatively investigated at the concentration of 100 μg ml^–1^. Interestingly, Kn2-7(K3R) shows the strongest hemolytic activity (89.3% hemolysis), followed by BmKn2-7R (73.1% hemolysis) (Figure 3B). The percent hemolysis of BmKn2-7 and BmKn2-7K were determined to be 41.0% and 12.2%, respectively (Figure 3B). Generally, Arg → Lys substitutions on the hydrophilic face of BmKn2-7 significantly reduce the hemolytic activity of the peptide (Figure 3B).

We then tested the antimicrobial activity of these peptides. As shown in Figure 3C, the MICs of these peptides against *S. aureus* ATCC29213 were in the range of 2.5–5 μg ml^–1^, which were more potent than those of *E. coli* ATCC25922 (MICs: 10–20 μg ml^–1^). However, no obvious variation was found between BmKn2-7, BmKn2-7K, BmKn2-7R and the other mutants, suggesting that Arg → Lys substitutions on the hydrophilic face of BmKn2-7 have no obvious impact on the antimicrobial activity of the peptide.

### BmKn2-7K Exhibits Potent Antimicrobial Actvity Against Antibiotic-Resistant ESKAPE Isolates

The above results indicate that BmKn2-7K retains the promising antimicrobial activity while has the lowest hemolytic activity among the tested peptides. To further evaluate its antimicrobial potential, MIC determination was performed against a series of clinically isolated antibiotic-resistant ESKAPE strains. For comparison, the MIC values of BmKn2, BmKn2-7 and BmKn2-7R were also measured. As shown in Table 3, these peptides exhibit potent and comparable activity against most of the tested gram-positive bacteria, including MRSA and MRSE, the MICs of the peptides against these bacteria strains were in the range of 2.5–5 μg ml^–1^. These peptides show good and comparable antimicrobial activity against *E. faecalis* and *E. faecium*, the MICs were determined to be 5–10 μg ml^–1^ (Table 3).

**TABLE 3:** Minimum inhibitory concentrations of BmKn2-7K against clinically isolated gram-positive pathogens.

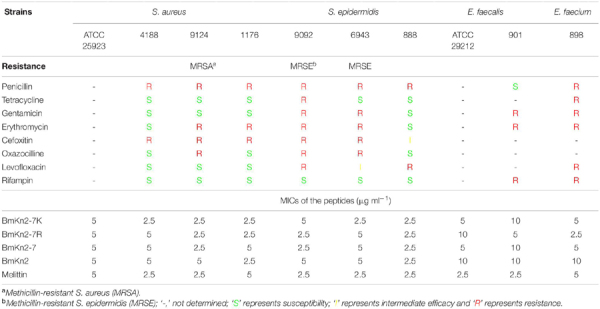

For the gram-negative bacteria tested, except for *A. baumannii* (MICs: 5–10 μg ml^–1^), the natural peptide BmKn2 is inactive to other tested gram-negative pathogens (Table 4). For ESBL-producing *E. coli*, CRE- and MDR *P. aeruginosa*, CRE- and MDR *A. baumannii*, BmKn2-7K and BmKn2-7R (MICs: 2.5–10 μg ml^–1^) showed potent and better antimicrobial activity than that of BmKn2-7 (MICs: 5–20 μg ml^–1^) (Table 4). All the three peptides showed potent and comparable antimicrobial activity against CRE *K. pneumoniae* (MICs: 10 μg ml^–1^) (Table 4).

**TABLE 4:** Minimum inhibitory concentrations of BmKn2-7K against clinically isolated gram-negative bacteria.

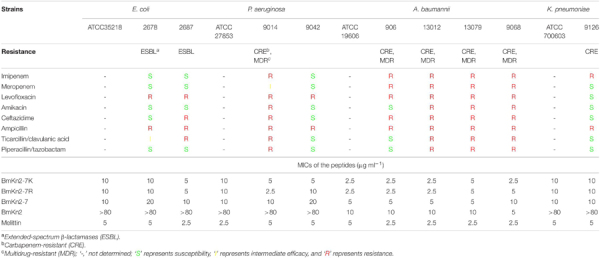

Taken together, these results demonstrated that Arg → Lys substitution has no significant influence on the antimicrobial activity of BmKn2-7, and suggest the potential of BmKn2-7K for combating antibiotic-resistant ESKAPE pathogens.

### Salt Sensitivity of BmKn2-7K

The peptide BmKn2-7K showed the lowest hemolytic activity among these peptides and exhibits potent antimicrobial activities against a wide range of ESKAPE pathogens. There are many kinds of cations in the physiological solution, so we further evaluated the effects of these cations on the antimicrobial activity of BmKn2-7K. As shown in Supplementary Table 1, for most of the tested bacterial strains, the addition of the cationic ions had no obviously negative effect on the antibacterial activity of the peptide.

### Antimicrobial Mechanism of BmKn2-7K

**FIGURE 4:**
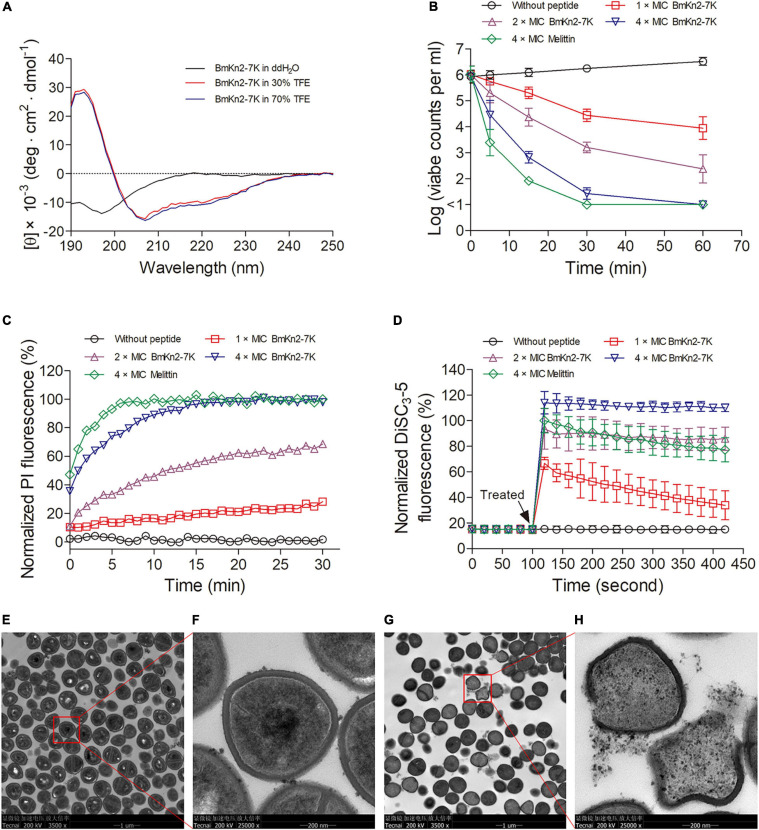
Antibacterial mechanism of BmKn2-7K. **(A)** CD spectra of BmKn2-7K (150 μg ml^– 1^) in ddH_2_O, 30% and 70% TFE solutions. **(B)** Time-killing kinetics of BmKn2-7K against *S. aureus* ATCC29213. The surviving bacterial cells were determined by counting the clonies on the agar after overnight incubation. **(C)** Normalized PI fluorescence of *S. aureus* ATCC29213 treated with BmKn2-7K. **(D)** Normalized DiSC_3_-5 fluorescence of *S. aureus* ATCC29213 treated with BmKn2-7K. In Figures 4B–D, the peptide concentrations were 0 × MIC (circle), 1 × MIC (square), 2 × MIC (upward triangle) or 4 × MIC (downward triangle). The sample treated with 4 × MIC melittin (diamond) was presented as the positive control. The data represent average ± standard error of three independent experiments. **(E,F)** Transmission electron microscopy (TEM) analysis of untreated *S. aureus* ATCC29213 and **(G,H)** the bacteria incubated with 1 × MIC BmKn2-7K for 0.5 h. **(F,H)** represent pictures of **(E,G)** (red box) with greater magnification.

Based on the above results, the mode of action of BmKn2-7K was investigated. As suggested by the previous reports (Fjell et al., 2011; Mahlapuu et al., 2016), the bacterial-killing process of CαAMPs generally starts with the formation of amphiphilic α-helix upon its adsorption to the bacterial membrane through electrostatic attraction, followed by the formation of various pores that led to membrane disruption, cellular metabolite leakage, and eventually bacterial death. To explore whether BmKn2-7K can fold into α-helix rich conformation, the secondary structure contents of the peptide was measured by CD spectroscopy. As shown in Figure 4A, BmKn2-7K exhibited only a negative peak centered at 198 nm in aqueous solution, indicating a randomly coiled structure; while the peptide exhibited a large positive peak centered at 195 nm and two negative bands centered at 208 and 222 nm in 30% or 70% TFE solutions, which mimics the hydrophobic environment in the bacterial plasma membrane (Cao et al., 2012). The results indicate that BmKn2-7K could fold into α-helical structure in the appropriate membrane environment.

Then, time-killing kinetics were determined against *S. aureus* ATCC29213 to evaluated whether BmKn2-7K has fast bacteria-killing ability. As shown in Figure 4B, the results demonstrated an increased killing rate with an increased BmKn2-7K concentration. Almost all of the bacteria were killed within 0.5 h as they were treated with 4 × MIC BmKn2-7K, and the sterilization curve was very close to that of bacteria incubated with 4 × MIC melittin, a representative membrane-targeting CαAMP that was used here as a positive control (Raghuraman and Chattopadhyay, 2007).

To determine whether BmKn2-7K can induce pore structure formation in the plasma membrane, PI uptake kinetics were determined. PI is a nucleic acid binding probe that can only permeate damaged plasma membrane, thus this probe was commonly used to evaluate the pore formation ability of AMPs (Mishra et al., 2019; Luo et al., 2021). As shown in Figure 4C, rapid and dose-dependent increases of PI fluorescence were observed upon the addition of BmKn2-7K, indicating that peptide treatment induced the formation of pore structures in the bacterial membrane. Peptide caused damage to the bacterial cell membrane was further evaluated by using the membrane potential sensitive probe DiSC_3_-5, this probe is quenched in intant bacterial cells, but it can be released out from the cells to produce fluorescence when the cytoplasmic membrane potential is disturbed (Toyomizu et al., 2002; Suzuki et al., 2003; Venkatesh et al., 2017). As shown in Figure 4D, when the bacterial cells were treated with BmKn2-7K, instantaneous and concentration-dependent increases of DiSC_3_-5 fluorescence were observed, indicating peptide-caused losses of membrane potential of the bacterial cells.

The mechanism of BmKn2-7K was also investigated by using TEM. Compared with untreated *S. aureus* (Figure 4E), blockage of bacterial division was observed in the BmKn2-7K-treated sample, and the number of intact bacteria in the sample treated with BmKn2-7K reduced dramatically (Figure 4G). Most importantly, the untreated bacteria had an intact morphology (Figure 4F), but obvious membrane fracture, ablation, and cell content leakage were observed in BmKn2-7K-treated bacteria (Figure 4H). Taken together, these results demonstrated that BmKn2-7K kills the bacteria *via* a membrane-lytic mechanism.

### Safety Profile of BmKn2-7K

**FIGURE 5:**
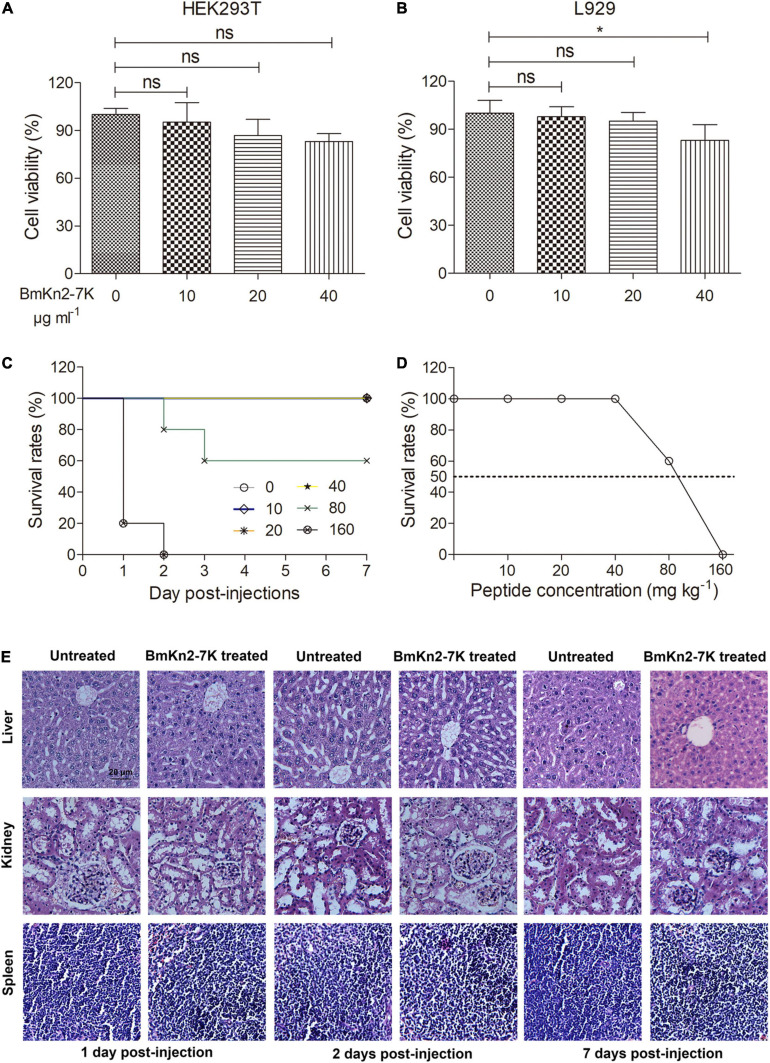
Safety profile of BmKn2-7K. **(A,B)** The cytotoxicities of the peptide were determined against HEK293T and L929. The data represent average ± standard error of three independent experiments. *P < 0.05; ns. represents “no significance.” The statistical significance between the groups was analyzed using one-way ANOVA followed by Tukey’s *post hoc* test to correct for multiple comparisons. **(C)** Kaplan-Meier plot for the survival of ICR mice treated with BmKn2-7K. **(D)** The survival rates of mice after treating with different doses of BmKn2-7K. Each cohort of ICR mice (*n* = 10) was injected intraperitoneally with a single dose of BmKn2-7K at concentrations ranging from 10 to 160 mg kg^–1^ body weight, followed by survival inspection for 7 days. Mice without peptide injection were set as the normal control. **(E)** Histopathological examination of ICR mouse liver, kidney and spleen sections. Each cohort of mice (*n* = 6) were injected intraperitoneally with a single dose of 40 mg kg^–1^ mouse body weight BmKn2-7K, and the mice were euthanized at 1, 2, and 7 days after treatment and stained with haematoxylin-eosin for observation. Mice without peptide injection were set as the normal control. The bar represents 20 μm.

To evaluate the *in vivo* toxicity of BmKn2-7K, the effects of BmKn2-7K on cell viability was firstly determined against the cells HEK293T and mouse fibroblast cells L929. The peptide was non-toxic to HEK293T at the concentration up to 40 μg ml^–1^ (Figure 5A), and only showed minor cytotoxicity to L929 cells at this concentration (Figure 5B). The *in vivo* toxicity of BmKn2-7K was determined in an healthy ICR mouse model (male, 6–8 weeks old, ∼30 g). As shown in Figure 5C, all mice injected with BmKn2-7K up to 40 mg kg^–1^ body weight survived for 7 days, 60% of the mice injected with BmKn2-7K at dose of 80 mg kg^–1^ body weight survived for 7 days, and all mice injected with BmKn2-7K at dose of 160 mg kg^–1^ body weight died within 24 h. These data indicate that the median lethal dose (LD_50_) of BmKn2-7K exceeds 80 mg kg^–1^ body weight (Figure 5D). To further evaluate the safety of the peptide, histopathological examinations of liver, spleen and kidney were performed in mice injected with BmKn2-7K at dose of 40 mg kg^–1^ body weight. As shown in Figure 5E, the tissues of liver, spleen and kidney displayed normal and intact morphologies at 1, 2, and 7 days post-treatment, and no necrosis or drug-induced injury was observed, demonstrating that BmKn2-7K is non-toxic at this dosage.

### *In vivo* Antimicrobial Efficacy of BmKn2-7K

**FIGURE 6:**
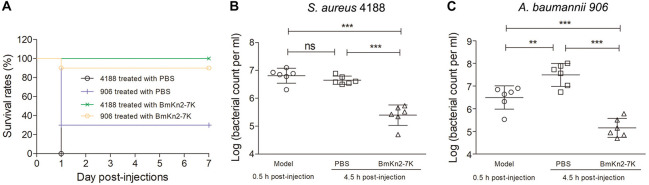
*In vivo* antimicrobial efficacy of BmKn2-7K. **(A)** Survival curves of infected mice after treatment with BmKn2-7K. Each cohort of ICR mice (*n* = 10) was infected with 5 × 10^7^ cfu of *S. aureus* 4188 (or 6.25 × 10^7^ cfu of *A. baumannii* 906) and cultured for 0.5 h to establish the lethal peritoneal infection model. Drug treatments were performed with a single dose of 20 (or 25) mg kg^– 1^ body weight BmKn2-7K for the *S. aureus* 4188 (or *A. baumannii* 906) infected model, respectively. **(B)** Quantitative determination of bacterial loads in the peritoneal fluid of *S. aureus* infected ICR mice (*n* = 6). **(C)** Quantitative determination of bacterial loads in the peritoneal fluid of *A. baumannii* infected ICR mice (*n* = 6). The logarithm value of the number of viable bacteria from each mouse was plotted as individual dots, error bars represent the standard deviation from the mean within each cohort, **P < 0.01; and ***P < 0.001; ns. represents “no significance”. The statistical significance between the groups was analyzed using one-way ANOVA followed by Tukey’s *post hoc* test to correct for multiple comparisons.

*Staphylococcus aureus* is one of the most notorious gram-positive bacterium that causes various lethal hospital-acquired infectious diseases (Chen and Huang, 2014; Dayan et al., 2016). To determine the *in vivo* antimicrobial efficacy of BmKn2-7K, ICR mice (male, 6–8 weeks old, ∼30 g) were inoculated intraperitoneally with 5 × 10^7^ cfu of penicillin-resistant *S. aureus* strain 4188 suspended in PBS to establish the mouse peritonitis-sepsis model. Treatment was performed at 0.5 h post-infection with a single intraperitoneal injection of BmKn2-7K (20 mg kg^–1^ body weight). The mice were monitored for 7 days. As shown in Figure 6A, all mice in the cohort treated with PBS (negative control) died within 24 h, but all mice treated with BmKn2-7K survived the study period and behaved normally. The antimicrobial efficacy was also evaluated by quantifying the bacterial load in the peritoneal fluid of the mice. As shown in Figure 6B, no significant variation of the bacterial load was observed in the cohort treated with PBS (5.73 × 10^6^ cfu ml^–1^ compared with 4.45 × 10^6^ cfu ml^–1^ at the starting point). However, the bacterial load in the cohort treated with 20 mg kg^–1^ body weight of BmKn2-7K decreased significantly to 2.73 × 10^5^ cfu ml^–1^ at 4 h post-treatment (*P* < 0.001).

The gram-negative bacteria *A. baumannii* has become a serious threat to public health worldwide due to the remarkable ability to survive in various healthcare environments and its increasing resistance to a wide range of antibiotics (Willyard, 2017; Wong et al., 2017). Therefore, we also investigated the *in vivo* antimicrobial efficacy of BmKn2-7K by using a mouse peritonitis model infected with CRE- and MDR- resistant *A. baumannii* 906 at dose of 6.25 × 10^7^ cfu. As shown in Figure 6A, only 30% of the mice survived one day after *A. baumannii* injection, but 90% of the mice which treated with BmKn2-7K at dose of 25 mg kg^–1^ body weight survived for 7 days and no abnormal behavior was observed for the surviving mice. The bacterial load in the peritoneal fluid was also determined. As shown in Figure 6C, the bacteria proliferated rapidly in the abdominal cavity of the mice within the study period, as the viable clones increased from 3.13 × 10^6^ cfu ml^–1^ (0.5 h post-infection) to 3.09 × 10^7^ cfu ml^–1^ (4.5 h post-infection) (*P* < 0.01). Compared to the group treated with PBS, the bacterial load in the cohort treated with 25 mg kg^–1^ body weight of BmKn2-7K dramatically decreased to 1.45 × 10^5^ cfu ml^–1^ at 4 h post-treatment (*P* < 0.001). Taken together, these results suggest that BmKn2-7K has good *in vivo* antimicrobial activity.

## Discussion

Cationic α-helical antimicrobial peptides are potential alternatives to traditional antibiotics for combating bacterial resistance, but bottlenecks such as strong hemolytic activity and *in vivo* inefficacy hinder their therapeutic development (Mahlapuu et al., 2016; Ghosh et al., 2019). Here, by investigating the two scorpion-derived peptides BmKn2 and BmKn2-7, we found that: i. keeping the hydrophobic face of BmKn2 unchanged, and increasing the number of alkaline residues (Lys or Arg) on the hydrophilic face reduces the hemolytic activity of the peptide and broadens its antimicrobial activity; ii. replacing Arg with Lys on the hydrophilic face significantly reduces the hemolytic activity of BmKn2-7 without influencing its antimicrobial activity; and iii: BmKn2-7K, in which all Arg residues were replaced with Lys on the hydrophilic face of BmKn2-7, displays the lowest hemolytic activity.

**FIGURE 7:**
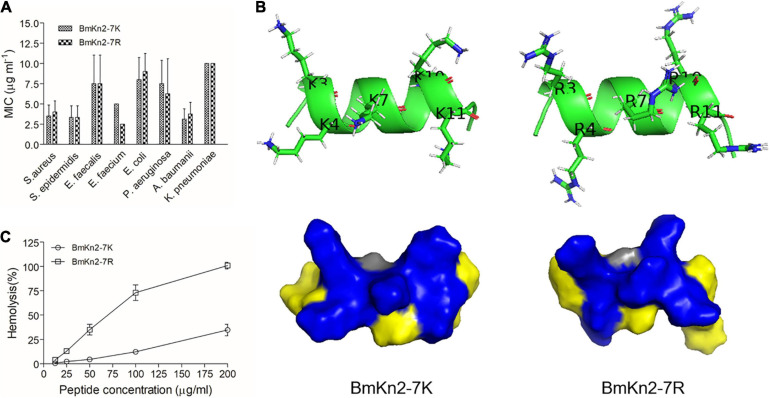
Structural and functional comparison of BmKn2-7K and BmKn2-7R. **(A)** No significant difference in the MIC values was observed between BmKn2-7K and BmKn2-7R. **(B)** Compared with BmKn2-7R, BmKn2-7K shows much lower hemolytic activity. **(C)** Compared with BmKn2-7R, BmKn2-7K shows a distinct structure surface view and side chain orientations on the hydrophilic face.

The plasma membrane of bacteria mainly consists of negatively charged lipids, including phosphatidylglycerol, cardiolipin, and phosphatidylserine; further, teichoic acid (gram-positive) and lipopolysaccharide (gram-negative) exist on the cell surface (Silhavy et al., 2010; Teixeira et al., 2012). This leads to a negative charge on the bacterial surface that can attract CαAMPs through electrostatic interactions (Fjell et al., 2011). Similar to other naturally occurring CαAMPs identified in scorpions, such as pantinins (Zeng et al., 2013) and lausporins (Zhao et al., 2021), the antimicrobial activity of BmKn2 is mostly restricted to gram-positive bacteria, such as *S. aureus* (Figure 2C). Interestingly, mutants such as Kn2(A4R) and Kn2(S10R), which were obtained by replacing Ala^4^ and Ser^10^ with Arg, respectively, showed good activity against gram-negative bacteria *E. coli* ATCC25922 (Figure 2C). On the other hand, no significant variation in antimicrobial activity was observed among BmKn2-7 analogs, such as BmKn2-7K and BmKn2-7R, both of which contain five alkaline residues (Figures 3C, 7A). Previous reports have shown that the antimicrobial activity of CαAMPs can be enhanced by replacing other residues with Lys or Arg, and this strategy is often employed in the designing of optimized peptides (Bessalle et al., 1992; Dou et al., 2017; Mourtada et al., 2019; Yang et al., 2019; Strandberg et al., 2020). Our study, consistent with these reports, suggested that electrostatic interactions between peptides and the cell surface of bacteria may be the critical determinant for their antimicrobial activity.

Many reports have also suggested that increasing the number of alkaline residues in the sequence of CαAMPs may be helpful to reduce their hemolytic activity, an increase in alkaline residues might lead to decreased hydrophobicity, which can attenuate the hydrophobic interactions between the peptide and the hRBC membrane (Dathe et al., 2001; Jiang et al., 2008; Li et al., 2016). By structural comparison, mutant design and function analyses, we demonstrated that while keeping the hydrophobic face unchanged, increasing the number of alkaline residues on the hydrophilic face of BmKn2 can reduce the hemolytic activity of the peptide (Figure 2B). However, significant variations in hemolytic activity were observed among the peptides, such as for Kn2(G3K_A4R), Kn2(G3K_S10R) and Kn2(A4R_S10R), which contain the same number of alkaline residues (Figure 2B). Our results further indicated a context-dependent and complicated profile of the effects of alkaline residues on the hemolytic activity of CαAMPs.

Perhaps the most striking finding in the present work is that the hemolytic activity of BmKn2-7K is much lower than that of BmKn2-7R (Figures 3B, 7B), which suggested that Lys and Arg play distinct roles in determining the hemolytic activity of CαAMPs. Natural CαAMPs are rich in Lys or Arg with an occurrence frequency of 9.52% and 5.86%, respectively (Wang et al., 2016), offering positively charged peptide characteristics (Bhunia et al., 2011; Gao and Zhu, 2018; Stone et al., 2019). In previous reports, proteins containing Lys or Arg at their active site can exhibit distinct physiological activity. This phenomenon has been found in cellular processes such as enzyme recognition, protein-RNA interaction and ion channel-toxin recognition (Wheatley and Holyoak, 2007; Berecki et al., 2014; Ukmar-Godec et al., 2019). However, to our knowledge, no study has explored the different roles of Lys *vs* Arg in CαAMPs. In our present work, we uncovered the distinct roles of Lys and Arg in determining the hemolytic activity of the CαAMP BmKn2-7. The disparity of Lys *vs* Arg in determining the hemolytic activity might result from their distinct interaction patterns. In fact, although both Lys and Arg are alkaline residues and carry the same charge (+1) at physiological pH, the positively charged groups at the terminus of the side chain of Lys or Arg have a distinct chemical nature. The side chain of Arg contains a guanidine group (p*K*a: 12.48), whereas the side chain contains an ε-amino group in Lys (p*K*a: 10.53). Lys can interact with other chemical group through methylene or ε-amino groups, the electron cloud is localized to the ε-amino group with weak directional preferences, whereas Arg contacts with other molecules through guanidine group, in which the electron cloud is delocalized over the planar guanidinium group with stronger directional preferences (Dougherty, 1996; Baud and Karlin, 1999), offering Arg the strong ability to interact with aromatic groups and form hydrogen-bonds (Gallivan and Dougherty, 1999; Kumar et al., 2018). Moreover, the modeled three-dimensional structures revealed distinct surface landscapes of BmKn2-7K and BmKn2-7R, and the side chain orientations of the charged residues on the hydrophilic face differ greatly (Figure 7C). On the other hand, the cell membrane of hRBCs consists of various kinds of zwitterionic lipids, i.e., phosphatidylethanolamine, phosphatidylcholine, sphingomyelin and cholesterol (Teixeira et al., 2012), which should be another important factor that influences CαAMP-hRBC interactions. Therefore, the mechanism underlying the different performances of BmKn2-7K and BmKn2-7R in hemolysis should be complicated, and further investigations will be necessary to address this directly.

BmKn2-7K is active against antibiotic-resistant ESKAPE bacteria, including MRSA, MRSE, ESBL-producing *E. coli*, CRE or MDR *P. aeruginosa*, CRE and MDR *A. baumannii*, and CRE *K. pneumoniae*, indicating that BmKn2-7K has broad-spectrum antimicrobial activity (Tables 3, 4). We also carried out various experiments to understand the bacterial killing mechanism of BmKn2-7K. BmKn2-7K kills bacteria quickly and induces rapid membrane disruption and cell content leakage (Figure 4). Most importantly, by using a mouse peritonitis model, BmKn2-7K was found to show potent antimicrobial efficacy not only against the clinically isolated penicillin-resistant gram-positive pathogen *S. aureus*, but also against CRE- and MDR-resistant *A. baumannii* and is non-toxic at antimicrobial dosages (Figures 5, 6). Taken together, our data indicate that BmKn2-7K seems to be a promising antimicrobial drug lead for future therapeutic development.

## Data Availability Statement

The original contributions presented in the study are included in the article/Supplementary Material, further inquiries can be directed to the corresponding author.

## Ethics Statement

The studies involving human participants were reviewed and approved by Human Welfare and Research Ethics Committee of Hubei University of Medicine. The patients/participants provided their written informed consent to participate in this study. The animal study was reviewed and approved by Animal Welfare and Research Ethics Committee of Xiangyang No. 1 People’s Hospital, Hubei University of Medicine (Permit number 2019DW002).

## Author Contributions

ZC designed the study and reviewed and edited the final version of the manuscript. XL, XY, WZhu, PY, ZZ, HG, and ZS performed the experiments. XL and ZC analyzed the data. LD, SL, and WZho contributed to analytic platform. MS and JW contributed to animal material. XL wrote the original draft of the manuscript. All authors contributed to the article and approved the submitted version.

## Conflict of Interest

The authors declare that the research was conducted in the absence of any commercial or financial relationships that could be construed as a potential conflict of interest.

## Footnotes

1
https://web.expasy.org/protparam/

2
https://heliquest.ipmc.cnrs.fr/

3
https://zhanglab.ccmb.med.umich.edu/I-TASSER/

## Supplementary Material

Supplementary Figure 1 HPLC profiles of synthesized BmKn2, BmKn2-7 and the other mutants.

Supplementary Figure 2 HPLC profiles of synthesized BmKn2-7K, BmKn2-7R and the other mutants.

Supplementary Table 1 Effects of physiological salts on the antimicrobial activities of the peptide BmKn2-7K.
