# Corrigendum: Fine-Tuning of Alkaline Residues on the Hydrophilic Face Provides a Non-toxic Cationic α-Helical Antimicrobial Peptide Against Antibiotic-Resistant ESKAPE Pathogens

**DOI:** 10.3389/fmicb.2021.815909

**Published:** 2021-11-30

**Authors:** Xudong Luo, Xiangdong Ye, Li Ding, Wen Zhu, Pengcheng Yi, Zhiwen Zhao, Huanhuan Gao, Zhan Shu, Shan Li, Ming Sang, Jue Wang, Weihua Zhong, Zongyun Chen

**Affiliations:** ^1^Institute of Biomedicine and Hubei Key Laboratory of Embryonic Stem Cell Research, College of Basic Medicine, Hubei University of Medicine, Shiyan, China; ^2^Hubei Key Laboratory of Wudang Local Chinese Medicine Research, Hubei University of Medicine, Shiyan, China; ^3^Department of Clinical Laboratory, Dongfeng Hospital, Hubei University of Medicine, Shiyan, China; ^4^Central Laboratory of Xiangyang No. 1 People's Hospital, Hubei University of Medicine, Shiyan, China; ^5^Department of Rehabilitation Medicine, Taihe Hospital, Hubei University of Medicine, Shiyan, China

**Keywords:** ESKAPE pathogens, antibiotic resistance, cationic α-helical antimicrobial peptide, hydrophilic face, lysine vs arginine, hemolytic activity, *in vivo* efficacy

In the original article, the Figure 6A, which shows the survival curves of infected mice after treatment with BmKn2-7K, was placed mistakenly using the same image as **Figure 5C**, due to a mistake made inadvertently in the preparation of the revised manuscript. The corrected Figure 6 appears below.

**Figure 6:**
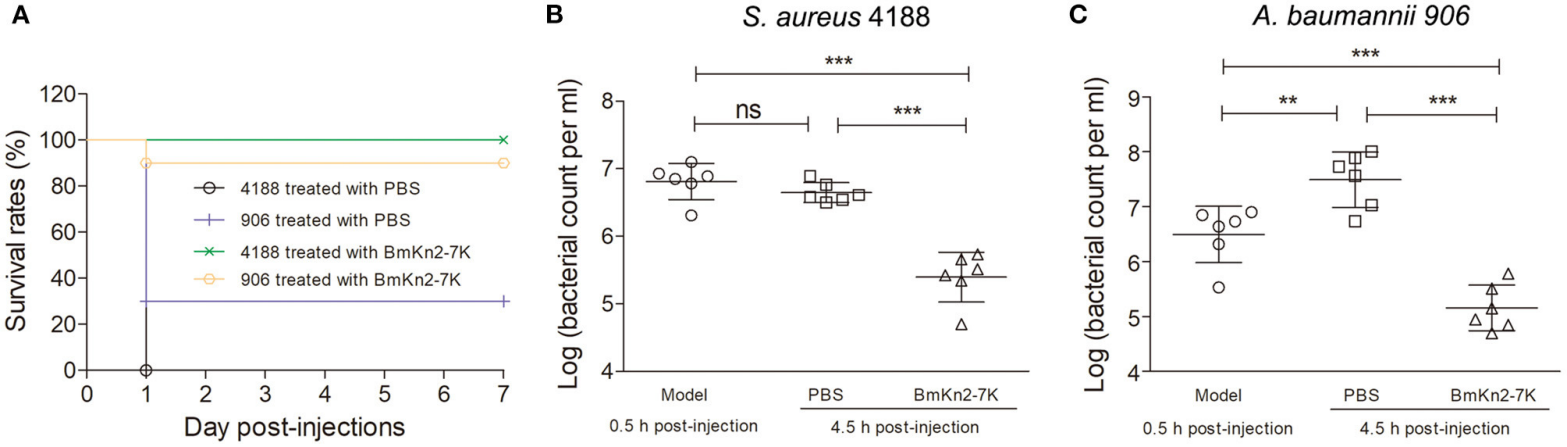
*In vivo* antimicrobial efficacy of BmKn2-7K. **(A)** Survival curves of infected mice after treatment with BmKn2-7K. Each cohort of ICR mice (*n* = 10) was infected with 5 × 10^7^ cfu of *S. aureus* 4188 (or 6.25 × 10^7^ cfu of *A. baumannii* 906) and cultured for 0.5 h to establish the lethal peritoneal infection model. Drug treatments were performed with a single dose of 20 (or 25) mg kg^−1^ body weight BmKn2-7K for the *S. aureus* 4188 (or *A. baumannii* 906) infected model, respectively. **(B)** Quantitative determination of bacterial loads in the peritoneal fluid of *S. aureus* infected ICR mice (*n* = 6). **(C)** Quantitative determination of bacterial loads in the peritoneal fluid of *A. baumannii* infected ICR mice (*n* = 6). The logarithm value of the number of viable bacteria from each mouse was plotted as individual dots, error bars represent the standard deviation from the mean within each cohort, ^**^P < 0.01; and ^***^P < 0.001; ns represents “no significance.” The statistical significance between the groups was analyzed using one-way ANOVA followed by Tukey's *post hoc* test to correct for multiple comparisons.

The authors apologize for the error and state that the correction has not changed the description, interpretation, or the original conclusion of the manuscript. The original article has been updated.

## Publisher's Note

All claims expressed in this article are solely those of the authors and do not necessarily represent those of their affiliated organizations, or those of the publisher, the editors and the reviewers. Any product that may be evaluated in this article, or claim that may be made by its manufacturer, is not guaranteed or endorsed by the publisher.

