# Supplementary material for: Fine-Tuning of Alkaline Residues on the Hydrophilic Face Provides a Non-toxic Cationic α-Helical Antimicrobial Peptide Against Antibiotic-Resistant ESKAPE Pathogens
Source: Front Microbiol. 2021 Jul 15;12:684591. doi: 10.3389/fmicb.2021.684591 (PMC8319832; doi:10.3389/fmicb.2021.684591)
Supplement: Supplementary Figure 1 — HPLC profiles of synthesized BmKn2, BmKn2-7 and the other mutants. [file Data_Sheet_1.DOCX]

**Supplemental Materials**

**Supplemental Table 1:** Effects of physiological salts on the antimicrobial activities of the peptide BmKn2-7K.

| Strains | MICs (μg ml^-1^) | | | | | | |
| --- | --- | --- | --- | --- | --- | --- | --- |
|  | Control | NaCl | KCl | CaCl_2_ | MgCl_2_ | NH_4_HCO_3_ | FeCl_3_ |
| *S. aureus* ATCC29213 | 5 | 5 | 5 | 5 | 5 | 5 | 5 |
| *E. faecalis* ATCC29212 | 5 | 10 | 20 | 10 | 20 | 20 | 20 |
| *E. coli* ATCC25922 | 10 | 10 | 10 | 10 | 10 | 20 | 20 |
| *P. aeruginosa* ATCC27853 | 10 | 10 | 10 | 10 | 10 | 20 | 20 |
| *K. pneumoniae* ATCC700603 | 10 | 5 | 10 | 10 | 10 | 10 | 10 |
| *A. baumannii* ATCC19606 | 2.5 | 2.5 | 5 | 2.5 | 2.5 | 5 | 5 |

The final concentrations of NaCl, KCl, CaCl_2_, MgCl_2_, NH_4_CO_3_ and FeCl_3_ were 150 mM, 4.5 mM, 2 mM, 1 mM, 6 μM and 4 μM, respectively. The control represents the samples without the addition of the salt ions.


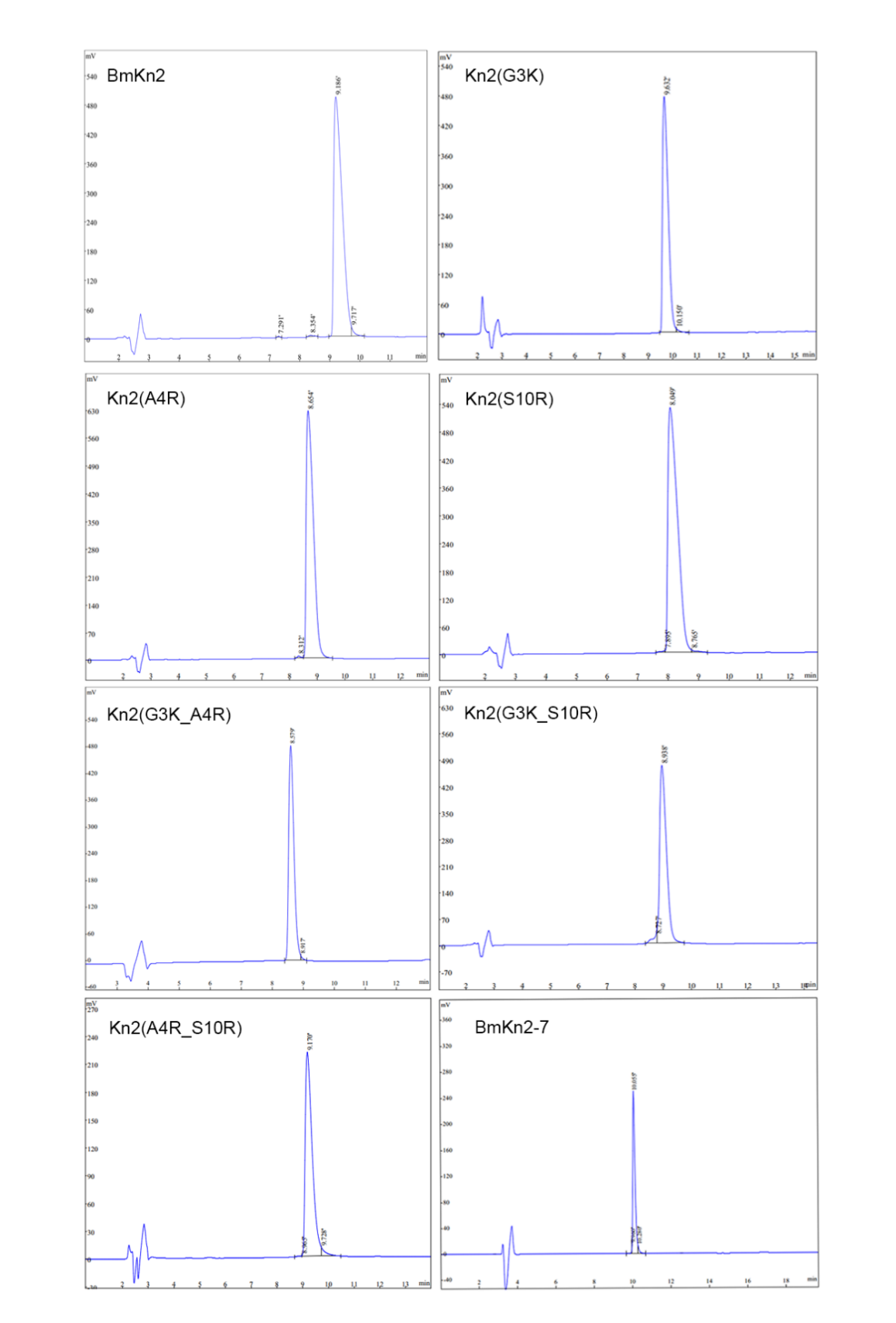
**Supplemental Figure 1.** HPLC profiles of synthesized BmKn2, BmKn2-7 and the other mutants.


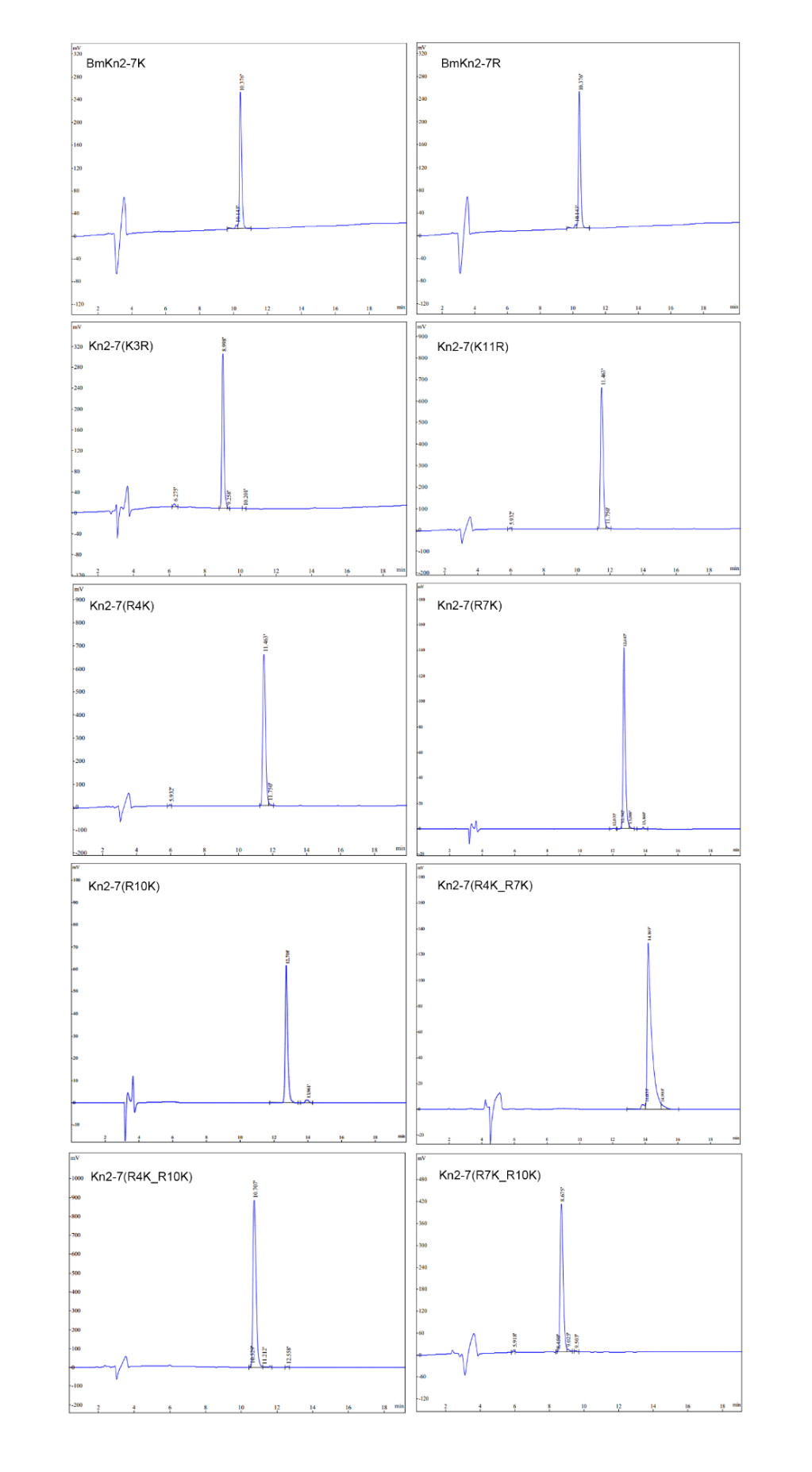
**Supplemental Figure 2.** HPLC profiles of synthesized BmKn2-7K, BmKn2-7R and the other mutants.
